# Efficient training protocol for rapid learning of the two‐alternative forced‐choice visual stimulus detection task

**DOI:** 10.14814/phy2.12060

**Published:** 2014-07-03

**Authors:** Shogo Soma, Naofumi Suematsu, Satoshi Shimegi

**Affiliations:** 1 Laboratory of Cognitive and Behavioral Neuroscience, Graduate School of Medicine, Osaka University, Toyonaka, Osaka, Japan

**Keywords:** Learning, Long–Evans rat, two‐alternative forced‐choice visual grating detection task, visual stimuli

## Abstract

The potential of genetically engineered rodent models has accelerated demand for training procedures of behavioral tasks. Such training is generally time consuming and often shows large variability in learning speed between animals. To overcome these problems, we developed an efficient and stable training system for the two‐alternative forced‐choice (2AFC) visual stimulus detection task for freely behaving rodents. To facilitate the task learning, we introduced a spout‐lever as the operandum and a three‐step training program with four ingenuities: (1) a salient stimulus to draw passive attention, (2) a reward‐guaranteed trial to keep motivation, (3) a behavior‐corrective trial, and (4) switching from a reward‐guaranteed trial to a nonguaranteed one to correct behavioral patterns. Our new training system realizes 1‐week completion of the whole learning process, during which all rats were able to learn effortlessly the association between (1) lever‐manipulation and reward and (2) visual stimulus and reward in a step‐by‐step manner. Thus, our new system provides an effective and stable training method for the 2AFC visual stimulus detection task. This method should help accelerate the move toward research bridging the visual functions measured in behavioral tasks and the contributing specific neurons/networks that are genetically manipulated or optically controlled.

## Abstract (precis)

We developed an effective and stable training system for the two‐alternative forced‐choice visual stimulus detection task, which introduced a spout‐lever as the operandum and a three‐step training program with four ingenuities: (1) a salient stimulus to draw passive attention, (2) a reward‐guaranteed trial to keep motivation, (3) a behavior‐corrective trial, and (4) switching from a reward‐guaranteed trial to a nonguaranteed one to correct behavioral patterns. This system realized 1‐week completion of the whole learning process.

## Introduction

Pigmented rodents have become a popular model in visual neurosciences (Laplante et al. 2005; Goard and Dan 2009; Kang and Vaucher 2009; Kang et al. 2013; Soma et al. 2013a,b), because the specific neurons of rodents are relatively easy to genetically manipulate or optically control (Adesnik et al. 2012; Olsen et al. 2012; Nienborg et al. 2013; Vaiceliunaite et al. 2013). These techniques have helped create a comprehensive network of visual information processing at the neuronal level. For example, Scanziani and his colleagues discovered in mice that the gain of a visual response to grating stimuli in the primary visual cortex (V1) is controlled by *layer 6* neurons (Olsen et al. 2012) and that the spatial summation tuning properties of excitatory V1 neurons is formed by somatostatin‐positive inhibitory neurons (Adesnik et al. 2012).

At the same time, there is increasing demand for quantitative measurement systems of the visual functions in behaving animals. Various visually guided behavior tasks including quantitative visual psychophysics have been applied to measure an animal's visual functions and corresponding neuronal substrates (Busse et al. 2011; Petruno et al. 2013). The two‐alternative forced‐choice (2AFC) visual stimulus detection task in freely behaving rodents is one example (Busse et al. 2011; Soma et al. 2013c). Recently, we measured the visual contrast detectability of freely moving rats by combining the 2AFC task and pharmacological administration of a cholinesterase inhibitor, finding that ACh improved the contrast sensitivity depending on the degree of difficulty of the stimulus detection (Soma et al. 2013c).

Previous studies examining visual functions commonly used gratings as the visual stimuli, because grating parameters such as orientation, spatial and temporal frequencies, contrast, and size can be independently controlled, making gratings suitable to examine not only the neuronal receptive field properties in early visual areas (Sengpiel and Vorobyov 2005; Osaki et al. 2011; Soma et al. 2012; Suematsu et al. 2012) but also the visual ability like the contrast sensitivity function of the animal (Birch and Jacobs 1979; McGill et al. 2004; Busse et al. 2011; Soma et al. 2013c). However, the learning of the visual discrimination or detection task using a grating stimulus requires a longer training period compared with a salient stimulus. For example, rats needed 7 weeks to learn the discrimination task for oriented grating (Meier et al. 2011), but only 1–3 weeks for high contrast figural stimuli such as symbols and statues (Bussey et al. 2008; Clark et al. 2011; Petruno et al. 2013; Reinagel 2013). This tendency is the same for the visual cue detection task. The task using the high contrast visual stimulus was learned in 2–4 days (Petruno et al. 2013; Reinagel 2013), but using the grating stimulus in about 2 weeks (Meier et al. 2011). Moreover, to measure perceptual detection limits, animals are required to perform the task at a psychological threshold (at low contrast in the case of a grating stimulus), which also needs a lengthy training period (Britten et al. 1992). Recently, we developed a training system for the 2AFC visual grating detection task in which rats were able to learn the paradigm within 2 weeks (Soma et al. 2013c). However, the actual number of days for the learning fluctuated largely between rats (6–12 days). Moreover, some rats failed outright to learn the paradigm (see fig. 2C in Soma et al. 2013c).

Here, we report a new and effective teaching method for the 2AFC visual stimulus detection task in which all rats can complete the learning within 1 week. The factors behind the success is ingenious training protocols wherein three‐step training enables rats to learn each process of the task in a step‐by‐step manner and each stage contains appropriate ingenuity to lead rats to the correct direction.

## Methods

### Ethical approval

All experimental protocols were approved by the Research Ethics Committee of Osaka University, and all procedures were carried out in compliance with the policies and regulations of the guidelines approved by the Animal Care Committee of the Osaka University Medical School and National Institutes of Health guidelines for the care of experimental animals.

### Subjects

Sixteen male Long–Evans rats (250–350 g; Institute for Animal Reproduction, Ibaraki, Japan) were kept on 12‐h light/dark cycles, and all training and testing were performed during the light phase. Six rats were used for the development of new methods (Figs. 2E and F, 3), and the remaining 10 rats were used for verification of the methods (Fig. 4).

### Water control

Rats had ad libitum access to water during weekends and obtained water only by performing the task correctly during the rest of the week. Signs of possible dehydration were monitored (reduced skin tension, sunken eyes, and marked variations in general behavior), but none were observed. To ensure adequate hydration, we weighed each rat at the beginning and end of each training day and compared the weight to a standard weight updated weekly. If the weight measured after the training was <90% the standard weight, the rat would be temporarily taken out of the study and given ad libitum access to water until the weight recovered. This condition never occurred in this study.

### Apparatus in the 2AFC visual stimulus detection task

The choice box (Fig. 2A; 30 cm long × 40 cm high × 55 cm wide) was made as described previously (Soma et al. 2013c) and is now commercially available from Narishige (EDMS13‐264; Tokyo, Japan). The front side of the box was translucent and faced a liquid crystal display (LCD) monitor. The box was divided by translucent walls to produce three connected areas that each had a spout‐lever in its center: a central lever in the middle area and choice levers in the other two. Rats could obtain the reward from a choice lever by pulling it upward. The reward volume was changed by controlling the open time of a solenoid valve that was manipulated by a PC. Speakers attached to the monitor gave signals indicating task initiation and auditory feedback of a task error (500 Hz). Rat behavior was monitored through a webcam. Software for the experimental control and stimulus presentation was written in MATLAB (Mathworks, Natick, MA) with extensions from the Psychophysics Toolbox (Brainard 1997; Pelli 1997; Kleiner et al. 2007).

### Three‐step method for teaching the 2AFC grating stimulus detection task

**Figure 1.**
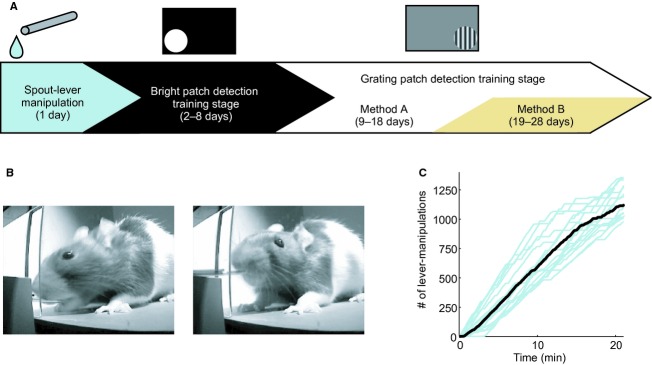
Training protocol and learning spout‐lever. (A) Schema of an effective method for teaching individual rats. First, rats acquire the reward from the spout‐lever. Next, they are trained to relate the reward to the blinking light patch (bright patch detection training stage). In this stage, they also learn how to start trials. After 1‐week training, the grating patch detection training stage is applied. In the experiments, although all rats understood the two‐alternative forced‐choice visual stimulus detection task during the bright patch detection training stage, some lost the association between the visual stimulus and reward during the grating patch detection training stage. In such cases, a switch from Method A to B was applied. (B) Photographs showing the rat pulling the spout‐lever up. (C) Number of lever‐manipulations on the first training day. All rats were able to pull the lever up (*n *= 16; pale blue lines). Black thick line shows mean data.

**Figure 2.**
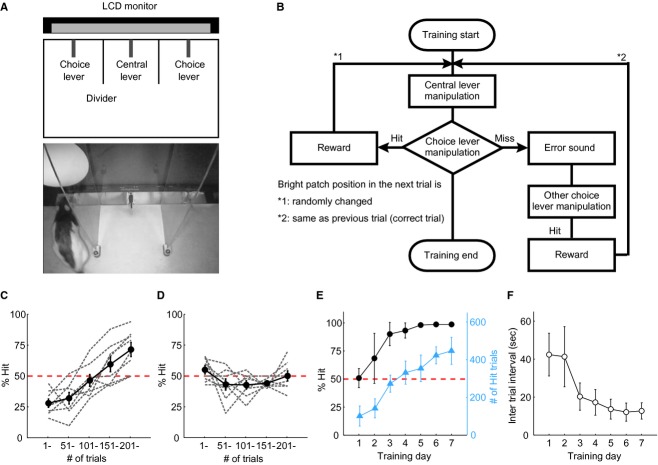
Training of bright patch detection. (A) Schema of the behavioral choice box and photograph of the bright patch detection training. (B) Flow chart of the two‐alternative forced‐choice (2AFC) visual stimulus detection task. Animals were required to detect the stimulus and pull the corresponding choice‐lever upward. When animals made a correct choice (Hit), water was delivered from the choice lever and the stimulus position in the next trial was changed randomly (*1). In the case of an incorrect choice (Miss), only an error sound was given, but the stimulus was still presented and rats obtained the water from the remaining choice lever (reward‐guaranteed). The next stimulus position was the same as that of the Miss trial (*2). (C–D) Learning curves on the first day of the second training stage. Two types of learning curves, improved (C; *n *= 8, sequenced choice) and flat (D; *n *= 8, random choice), were observed. These curves reveal differences in behavioral strategies for the same task (details are described in the text). (E–F) Population learning curves in the bright patch training stage (*n *= 6). Animal learning of the 2AFC task paradigm rapidly progressed within 3–4 training days, as indicated by the rapid increase in both % Hit (E; black circles) and the number of Hit trials (E; blue triangles), and decrease in intertrial interval (F). Dashed lines show chance level (50% Hit). Data are presented as mean ± SEM.

**Figure 3.**
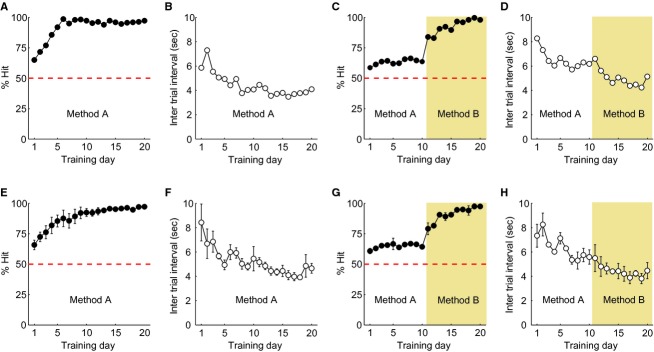
Training of grating patch detection: Methods A and B. (A–D) Typical learning curves of % Hit and intertrial interval (ITI) in a quick (A–B) and slow learner (C–D). The quick learner adapted a strategy in which it learned continuously from the bright patch detection training stage to the grating patch detection training stage (A–B). In contrast, the performance of the slow learner did not improve until the researcher changed the method (C–D). Once the training was conducted under Method B, the performance rapidly improved. (E–H) Population functions of % Hit and ITI in quick (E–F; *n *= 4) and slow learners (G–H; *n *= 2). Data are presented as mean ± SEM. Dashed lines show chance level.

Rats were trained in three stages for the 2AFC grating stimulus detection task (Fig. 1A). In the first stage, all rats (*n *= 16) learned to obtain fluid delivered from the spout‐lever by pulling the choice lever up (Fig. 1B and C). In the second stage, the bright patch detection training stage, the rats learned the basic procedure of the 2AFC task, that is, how to initiate a trial and how to obtain fluid in a normal choice box (Fig. 2A). In the third stage, the grating patch detection training stage, rats learned that the fluid supply was associated with a grating patch during which two different training protocols, Methods A and B, were applied depending on the rat's behavioral patterns. To evaluate the time course of the rat's behavior (*n *= 6), the training was continued for 20 days despite completion of the task learning (Fig. 3). Through these training periods, rats learned the following in a stepwise fashion: association between (1) the reward and lever‐manipulation and (2) the reward and visual stimulus.

#### First stage: spout‐lever manipulation

In the first stage, rats were placed in a single choice box isolated from the other areas to learn the association of lever‐manipulation with the fluid (Fig. 1A and B). First, rats were habituated to the new environment in 10 min, during which the fluid was delivered from the choice lever automatically every 30 s. After the habituation phase, rats were returned to their home cage for 30 min to freely move about and then placed in the same choice box for 20 min, but with the fluid being delivered only if the lever was pulled up. In this study, we adopted the spout‐lever integrating a lever (operandum) and a rewarding spout (reinforcer) to facilitate the operant learning (Kimura et al. 2012; Soma et al. 2013c, 2014). All rats successfully learned to obtain the reward by pulling the choice lever up within 1 day (Fig. 1C).

#### Second stage: bright patch detection training

In the second stage, rats learned the basic procedure of the 2AFC task including how to initiate a trial and how to obtain the reward in the normal choice box (Fig. 2A). A blinking bright white patch (75 cd/m^2^; temporal frequency, 3 Hz; diameter, 70°) was presented on the LCD monitor (background luminance, 1 cd/m^2^) by pulling the central lever upward, and the visual stimulus presentation continued until rats pulled upward the correct choice lever by which fluid was delivered. When any choice lever was pulled upward before the central lever, only an audible sound was given as an instructive feedback signal. When rats chose the incorrect choice lever (Miss) after the central lever, the same audible sound was given and the visual stimulus was still presented (Fig. 2B). Therefore, rats could eventually access the fluid by choosing the other choice lever even in the Miss trial. The task result determined the position of the visual stimulus in the next trial. In the case of a Miss trial, the position of the visual stimulus in the next trial was the same as the previous trial (behavior‐corrective trial). In the case of a Hit trial, the visual stimulus was presented pseudorandomly on either side. Each session continued for 60 min. At least two training sessions per day were conducted for 1–2 training days of the second stage. On the remaining training days, rats were trained in at least one session per day.

#### Third stage: grating patch detection training

**Figure 4.**
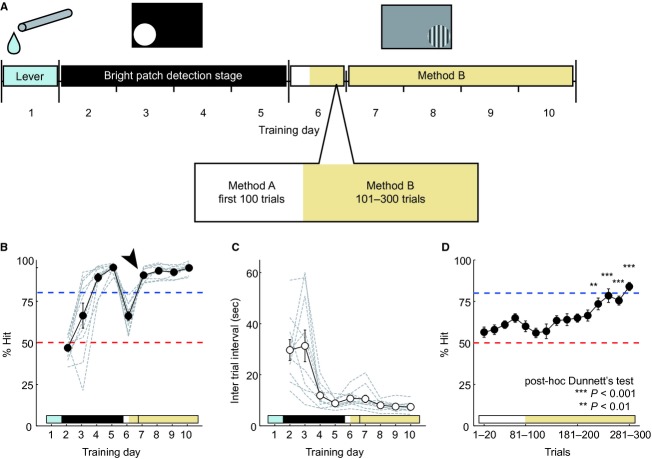
Optimal protocol for teaching the two‐alternative forced‐choice (2AFC) grating detection task. (A) Schema of the most stable and efficient method for learning the 2AFC grating detection task. On the first training day, rats learned the lever‐manipulation (1 day, Lever). After 4‐day training of the bright patch detection stage (2–5 days), the grating patch detection stage was applied (6–10 days). On the 6th training day, rats were trained first with 100 trials under Method A and then 200 trials under Method B. (B–C) Learning curve of % Hit (B) and intertrial interval (C) at the population level obtained from ten rats. Arrowhead shows the completion of learning within 1 week. (D) The learning curve on the transition day (6 days) to Method B from Method A. Some rats showed low % Hit in the first 100 trials, but all rats learned the task before the day was done. Data are presented as mean ± SEM. Red dashed lines show chance level. Blue dashed lines show the criteria for learning completion (80% Hit).

In the third stage, rats were trained to learn that the grating stimulus was associated with reward by one of two protocols, Method A or Method B, depending on the rat's behavioral patterns. In both methods, the mean luminance of the LCD monitor was 30 cd/m^2^ and a stationary vertical grating patch (spatial frequency, 0.1 cycles/degree; diameter, 70°) was presented pseudorandomly on the right or left side of the LCD monitor. The third stage was basically the same as the procedures used in the second stage except for a stationary vertical grating patch being used as the visual stimulus. The reward was guaranteed even in a Miss trial for Method A, but not for Method B. This training stage is indicated by the pale yellow areas in Figures 1, 3, and 4. Each session continued for 30 min, and at least two sessions were conducted on day 1 of the third stage.

### Data analysis

Rat performance (% Hit) was calculated by dividing the number of correct trials by that of total trials. In the bright patch detection training stage and Method A in the grating detection training stage, the incorrect lever‐manipulation data before manipulation of the correct choice lever was recorded. Therefore, we were able to calculate % Hit in these two training stages. In these stages, the results obtained from the behavior‐corrective trial were purposely included for the % Hit calculation to reflect the difference of the rats’ behavioral strategies to the % Hit. For example, the % Hit fell below chance level for the rats taking a sequenced choice and came close to chance level for a random choice (see Results).

### Statistical analyses

The one‐way analysis of variance (ANOVA) followed by post‐hoc Dunnett's test was used to compare % Hit obtained from the first 20 trials before the switch of the training method with that after the switch (Fig. 4D).

## Results

We first describe our three‐step method for the development of an efficient 2AFC grating stimulus detection task step by step, and then demonstrate the optimal protocol established on the basis of our results.

### Learning spout‐lever manipulation

Rats were placed in a single choice box isolated from the other areas to learn the association of the lever‐manipulation with the fluid. By chewing on the tip of the spout‐lever (left picture in Fig. 1B) and pulling it up, rats could acquire the reward directly from the tip (right picture in Fig. 1B). The difficulty in this learning process is to awaken animals to the specific behavior associated with the reward delivery. The spatial dissociation between the operandum and reward supplier or the temporal dissociation between the animal's behavior and reward acquisition makes it difficult for the animals to notice the association. The spout lever solves these problems (see fig. 1B in Kimura et al. 2012).

In the first stage of our training, the rats learned to explore the choice box and obtain fluid delivered from the tip of the choice lever automatically every 30 s. Rats were strongly motivated by using the lever as a reward supplier during this period, and thereafter rats reached out actively to touch and manipulate the lever. All rats learned how to manipulate the spout‐lever within 20 min on the first day of the first stage (Fig. 1C). This stage was necessary for teaching the trial initiation in the following bright patch detection training stage.

### Training of bright patch detection

In the second stage, rats learned two things: (1) the basic procedures of the 2AFC task, such as how to initiate the trial and how to obtain the reward, and (2) the association of the reward with bright patch detection. Figure 2A shows the choice box for the 2AFC visual stimulus detection task. The first difficulty in this learning process is that animals are not easily aware of the visual stimulus. To overcome this issue, we made high contrast in luminance between the visual stimulus and background on a LCD monitor, where a white blinking patch stimulus (bright patch, 75 cd/m^2^) was presented on a black background (1 cd/m^2^).

The second difficulty is that, in general, rats tended to capitulate pulling up the lever under hard to obtain reward situations when they repetitively failed to obtain the reward. Therefore, we adopted the reward‐guaranteed task shown in Figure 2B, in which rats could constantly obtain the fluid in a trial independently of their lever choice. Rats pulled the central lever upward to initiate the task, which triggered the presentation of a bright patch on the right or left side of the LCD monitor, and then pulled upward the choice lever corresponding to the stimulus to obtain a reward (Hit). In the case of Hit, rats received a reward of 5–10 μL of water, and the stimulus position in the next trial was determined at random (*1 in Fig. 2B). When rats made an incorrect choice (Miss), rats received an audible sound only, and the stimulus was presented until the rats obtained the reward by pulling up the correct choice lever. This protocol proved very effective at maintaining the animal's motivation toward lever‐manipulation.

However, the reward‐guaranteed task brought another problem, as some rats would learn a specific sequence for pulling the three levers (sequenced choice). An example sequence could be pulling up the central lever first, then the right choice lever second, and the left choice lever third. The challenge was to overcome this problem without compromising rat motivation. Since rats were eventually able to obtain a reward using a sequence‐dependent choice strategy, they did not perceive a need to change strategy. If the stimulus position is randomly determined for each trial, rats can make a correct choice at chance level (50% Hit) by the patterned strategy. To explicitly remind them that another behavioral strategy is better than the sequenced choice strategy, the stimulus was presented on the same side as a Miss trial (*2 in Fig. 2B; behavior‐corrective trial). Thus, animals were easily able to notice that the reward is delivered by another nonpreferred choice lever, which facilitates the shift of their preference from a certain choice lever to another one. The effectiveness is clearly shown in the rapid raise of the learning curve of the rats (*n *= 8) for the sequence‐dependent choice on the first training day (Fig. 2C). Since such rats initially repeated the same incorrect choice despite the stimulus being presented on the same side by the behavior‐corrective trial, their task performance (% Hit) in the first 50 trials fell below chance level. However, their performance rapidly improved thereafter, indicating that the behavior‐corrective trial is very effective at promptly modifying behavior and at quantifying the behavior strategy.

Another type of behavioral strategy was observed in eight rats (Fig. 2D). Here, % Hit generally continued to be chance level, suggesting that the rats made their choice at random (random choice). The performance of these rats was not corrected until 250 trials on the first training day (Fig. 2D), but improved smoothly thereafter (see Fig. 2E). Figure 2E and F show time courses of the averaged learning curves of the rats (*n *= 6) with the sequenced choice and random choice strategies in % Hit, the number of Hit trials per day, and intertrial interval (ITI), respectively. All rats were able to learn the basic procedures of the 2AFC visual detection task within 3–4 days of the bright patch detection training stage (Fig. 2E and F). The % Hit was rapidly improved from 50% (chance level) to more than 80% within 3 days in parallel with the number of Hit trials (Fig. 2E), and ITI was rapidly shortened from 40 to 20 sec within the same period (Fig. 2F). These results clearly demonstrate that the behavior‐corrective trial is effective at modifying behavior regardless of the behavioral choice and that the combination of a highly salient stimulus and reward‐guaranteed task with a behavior‐corrective trial enabled rapid and efficient learning of the 2AFC task.

### Training of grating patch detection: Methods A and B

The final training stage started with Method A and was switched to Method B as needed. In Method A, a visual stimulus was simply replaced from the bright patch to the vertical grating patch. Four of six rats were able to generalize the procedures of the 2AFC acquired from the second training stage and applied them to the new grating stimulus (Fig. 3A and E). The other two rats could not (Fig. 3C and G). The former and latter animals were called quick and slow learners, respectively. Typical examples of a quick learner for learning curves of % Hit and ITI are shown in Figure 3A and B, respectively. % Hit exceeded chance level from day 1 and gradually improved until day 6 (Fig. 3A). ITI also gradually decreased during the same days (Fig. 3B). The same tendency was observed at the population level (Fig. 3E and F, *n* = 4).

Figure 3C and D show an example of a slow learner. This animal did not improve its task performance even after 10 days of training under Method A, while % Hit remained slightly above chance level (dashed line in Fig. 3C). Therefore, slow learners needed an additional reinforcer to explicitly notice which choice was an error. Thus, Method A was switched to Method B. The difference between the two methods was that the reward was guaranteed for Method A but not for Method B. Absence of a reward acted as strong negative reinforcement, which is evidenced by the fact that once the slow learner was trained in Method B its performance was rapidly and dramatically improved (Fig. 3C). The slope of the learning curve after this switch was steeper than the slope of the quick learner using Method A (Fig. 3A). Population data (*n *= 2) agreed with the data of the one slow learner, indicating that switching from Method A to Method B facilitated rapid learning (Fig. 3G and H). On the other hand, introducing Method B from the start of the third stage was ineffective, because animals were confused by the simultaneous change in two factors, stimulus type and reward‐guarantee, compromising their motivation to apply the trial and error strategy and quit the task (data not shown).

Unlike % Hit, ITI decreased with training days for both quick and slow learners, showing a steep curve in early training days and subsiding later (Fig. 3B, D, F, and H). This property indicates that the speed of the task execution depends strongly on the number of trials. Additionally, switching Method A to B shortened the ITI for slow learners (Fig. 3D and H).

### Optimal protocol for teaching the 2AFC grating detection task

By adopting the above‐mentioned procedures, we developed a stable and efficient method for teaching the 2AFC grating detection task to rats. Figure 4A shows the scheme of the teaching program. First, rats are taught how to manipulate a lever and how to obtain a reward using the spout‐lever on day 1. Second, rats are taught the basic procedures of the 2AFC visual stimulus detection task and the association between a stimulus and corresponding choice lever over a 4‐day training period that combines a salient bright stimulus and reward‐guaranteed task with a behavior‐corrective trial. Finally, rats are taught the 2AFC grating detection task using Methods A and B.

Ten rats were trained according to our teaching program. The population learning curves are shown in Figure 4B–D. Figure 4B and D show changes in % Hit over the training days and over the trials on training day 6 (day 1 of the third stage), on which day they were trained with 100 trials using Method A and then with 200 trials using Method B. The switch from Method A to B significantly improved % Hit (one‐way ANOVA, *F*_14,60_ = 7.2, *P *< 0.001; post‐hoc Dunnett's test, 221*–*240 trials, *P *< 0.01, 241*–*300 trials, *P *< 0.001; Fig. 4D). All rats learned the 2AFC visual stimulus detection task stably and effectively within a week, indicating that our teaching program is applicable to animals that use different behavioral strategies.

## Discussion

The 2AFC visual stimulus detection task can be used for various research purposes including the measurements of visual functions (Busse et al. 2011; Meier et al. 2011; Petruno et al. 2013; Soma et al. 2013c), the modeling of decision making (Busse et al. 2011; Meier and Reinagel 2011; Carandini and Churchland 2013), and the assessment of memory retrieval functions (Soma et al. 2014). However, its application is hindered by the time it takes to train the animals. We therefore established a stable and efficient training system for freely moving rats. Our new training system realized 1‐week completion of the whole learning process in all tested rats (arrow head in Fig. 4B).

The stability and efficiency of our training system are supported by both ingenious hardware and software elements on the system. The specific hardware element is the spout‐lever. In previous studies that used operant conditioning, animals first had to learn how to manipulate the operandum, but obtained the reward elsewhere (Adams 1982; Coutureau and Killcross 2003; Yin et al. 2004). The spout‐lever integrates these steps into one, which enables rapid learning of the relationship between lever‐manipulation and the reward (Kimura et al. 2012; Soma et al. 2013c, 2014). This hardware shortened the total number of training days and motivated the animals to manipulate the lever during the whole learning process.

The most important factors for enabling highly stable and efficient animal learning were software elements that responded to the animal behavior. When and how the behavior is reinforced can have a dramatic impact on the strength and rate of the responses. We implemented four ingenuities into our teaching program: (1) a highly salient bright stimulus contrasted with a black background, (2) a reward‐guaranteed trial, (3) a behavior‐corrective trial, and (4) switching of the training procedures from a reward‐guaranteed trial (Method A) to a reward‐nonguaranteed trial (Method B).

Our previous teaching system incorporated the spout‐lever but not any of the above ingenuities (Soma et al. 2013c). It took about 2 weeks to train the animals, but large variation was seen between animals (6–12 days), and even then some never did learn the 2AFC grating detection task paradigm. Our new teaching program, on the other hand, shortened the training period to 1 week, had far less variability between animals in learning time, and succeeded in teaching all animals.

### Factors that enhance the efficiency and stability of animal training

Attention is an important factor that facilitates the speed of learning the visual stimulus detection 2AFC task. The white bright stimulus highly contrasted with black background used in the second stage is a highly salient and effective way to draw the animal's passive attention. Attention is closely related with cognitive function as well as memory function, and selective attention acts as a filter through which the attended sensory information is selectively transferred from the sensory register as an instant memory store to a short‐term memory system. Sensory information not represented in short‐term memory is thought to be instantly lost and never stored as long‐term memory. Therefore, drawing an animal's attention is crucial for associative learning when using a visual stimulus. In fact, recent studies using salient stimuli have also achieved short‐term learning (within 2 weeks) of the visual cue detection task (Petruno et al. 2013; Reinagel 2013). Thus, an attention‐getting salient visual cue is useful for teaching the basic procedure of the 2AFC task before introducing the sinusoidal gratings.

The operant learning is based on a contingent reinforcement, and an appropriate combination and timing of reinforcers facilitate efficient and stable animal learning. The reinforcers in the present teaching program kept the animal motivated until completion of the learning and induced the animal to switch its behavior promptly. In general, animals were confused by a new training condition, for example, when they moved to the next step of a training program. In this study, there were two stage transitions: the first to second stage and second to third stage. In both cases, the rats needed to learn a new rule by taking a trial and error strategy. The accidental behavior–reward contingency had no small effect on motivation and subsequent behavior. When rats were trained by a standard procedure for the 2AFC task at the beginning of a new stage, that is the reward was provided only following the correct lever choice, some rats quit the task after a process of trial and error. There was a tendency for rats that accidentally repeated the incorrect choice to abandon the task due to poor motivation (data not shown). To prevent this loss of motivation, a constant positive reinforcement with reward‐guaranteed trial was found to work effectively. Accordingly, we applied reward‐guaranteed trials in the second training stage and at the beginning of third training stage.

However, reward‐guaranteed trials had disadvantages as well. Some rats repeated a fixed order of lever choice independent of the visual stimulus. For example, a certain rat preferentially chose the right choice lever (preferred lever) and then the left choice lever (nonpreferred lever) regardless of the stimulus. Because rats could not fail to obtain the reward, no association between the first lever choice and reward was made. To correct the behavioral strategy, we introduced a behavior‐corrective trial (Fig. 2B). When rats chose the incorrect choice lever, the visual stimulus of the next trial was presented at the same location as the Miss trial. Thus, rats always obtained the reward from the nonpreferred lever, which would be the left lever in the above example. Consequently, the behavior toward the nonpreferred lever is reinforced (positive reinforcement) and that toward the preferred lever is weakened (negative reinforcement). This combination of reward‐guaranteed trial and behavior‐corrective trial is of considerable benefit for correcting behavior without reducing rat motivation.

The timing of the switch from the reward‐guaranteed trial (Method A) to a standard 2AFC trial (Method B; reward‐nonguaranteed trial) is important, because it causes both positive and negative effects depending on the animal's level of achievement. Since a first experience for no reward confuses an animal, a high probability of Miss trials diminishes motivation before the association of the visual stimulus with reward is sufficiently learned. In this study, % Hit was about 90% on the last day of the second training stage, and the performance on the first day of the third training stage was deteriorated but still larger than chance level (50% Hit). This result suggests that the relationship was well learned and that stimulus generalization was already in progress. Therefore, the trial switch successfully accelerated learning by adding a negative punishment at the appropriate moment.

### Utility and significance of our rodent training system for visual tasks

Recent advances of electrophysiological, transgenic, and optogenetic techniques on behaving rodents have enabled us to examine the neural correlates of specific behaviors by recording and intentionally changing the neuronal/network activities. To understand exactly the neuronal mechanisms underlying an animal's natural behavior, it is important that the experimental behavioral conditions resemble natural conditions as much as possible, as otherwise task‐irrelevant factors may affect the results. The most important merit of our training program is for the rodents to learn the task and to undergo the test in a natural and less‐stressful situation, by which they conduct the task voluntarily and independently. We designed the system to eliminate various kinds of stressors, including any stress caused by restraining the head or body, and psychological stress such as fear or anxiety due to electrical shock, water immersion, or uncomfortable repeated handling by humans. We also minimized water restriction under a 90% weight requirement plus ad libitum access 2 days per week, whereas in previous studies, animals were often restricted to 80–85% of their feeding weight and received a controlled amount of water 7 days per week (Busse et al. 2011; Histed et al. 2012; Glickfeld et al. 2013). Therefore, our system is expected to help extract pure neural correlates with specific behaviors under a stress‐free situation.

The visual tasks in the 2AFC paradigm allow us to study not only visual functions but also other higher order functions such as decision making (Busse et al. 2011; Meier and Reinagel 2011; Carandini and Churchland 2013) and memory (Soma et al. 2014). Since the basic concepts of our system can be applied to the teaching of tasks beyond visual ones, our training method is expected to be applicable to a wide range of research fields.

## Acknowledgments

We thank Drs Yoshikazu Isomura, Yutaka Sakai, Hajime Sawai, Tomomitsu Miyoshi, and Takashi Kitsukawa for discussions and comments. We also thank Yuka Yamamoto for technical help, and Dr. Peter Karagiannis for improving the English of the manuscript.

## Conflict of Interest

There are no conflicts of interest to disclose by the author.
